# Supplementary material for: P-TRAP: a Panicle Trait Phenotyping tool
Source: BMC Plant Biol. 2013 Aug 29;13:122. doi: 10.1186/1471-2229-13-122 (PMC3848748; doi:10.1186/1471-2229-13-122)
Supplement: Additional file 7 — P-TRAP source code. The Netbeans project that contains the source code of the application. [file 1471-2229-13-122-S7.zip › javadoc.zip!/javadoc/org-biolag-particleprocessor/index-all.html]

Index (ParticleProcessor)


JavaScript is disabled on your browser.


- Package
- Class
- Tree
- Deprecated
- Index
- Help

*org.biolag.particleprocessor 1.0*

- Prev
- Next

- Frames
- No Frames

- All Classes

A C E F G I M N O P R S T V W 


## A

addConcavePoint(ConcavePoint) - Method in class org.biolag.particleprocessor.Particle


addContourPoint(Point) - Method in class org.biolag.particleprocessor.Particle


area() - Method in class org.biolag.particleprocessor.Particle


## C

calcAll() - Method in class org.biolag.particleprocessor.ParticlesModel
:   Calculate all traits.

calcArea(ArrayList<Particle>) - Static method in class org.biolag.particleprocessor.ParticlesAnalyizer


calcAreaFromContour(ArrayList<Particle>) - Static method in class org.biolag.particleprocessor.ParticlesAnalyizer


calcBoundingBox(ArrayList<Particle>) - Static method in class org.biolag.particleprocessor.ParticlesAnalyizer


calcBounds(ArrayList<Particle>) - Static method in class org.biolag.particleprocessor.ParticlesAnalyizer


calcCircularity(ArrayList<Particle>) - Static method in class org.biolag.particleprocessor.ParticlesAnalyizer


calcCompactness(ArrayList<Particle>) - Static method in class org.biolag.particleprocessor.ParticlesAnalyizer


calcConcavePoints(ArrayList<Particle>, AImage, int) - Static method in class org.biolag.particleprocessor.ParticlesAnalyizer
:   calculate the concave points using a 5x5 window centered on the contour point and it is neighbors
    and the outcome is the summation of the contour points in these three windows

calcContour(ArrayList<Particle>, AImage) - Static method in class org.biolag.particleprocessor.ParticlesAnalyizer
:   Find the contours of the particles using math morphology

calcContour2(ArrayList<Particle>, AImage) - Static method in class org.biolag.particleprocessor.ParticlesAnalyizer
:   a slow but more accurate method to get the contour of the particles

calcCovixity(ArrayList<Particle>, AImage, int) - Static method in class org.biolag.particleprocessor.ParticlesAnalyizer
:   Convexity measure based on Prof.Dr.

calcCurves(ArrayList<Particle>, double) - Static method in class org.biolag.particleprocessor.ParticlesAnalyizer
:   converts a given particle into two sets of concave and convex curves

calcEllipticity(ArrayList<Particle>) - Static method in class org.biolag.particleprocessor.ParticlesAnalyizer


calcHeighAndWidth(ArrayList<Particle>) - Static method in class org.biolag.particleprocessor.ParticlesAnalyizer


calcNetArea(ArrayList<Particle>) - Static method in class org.biolag.particleprocessor.ParticlesAnalyizer
:   calculating the area using the green equation

calcPosition(ArrayList<Particle>) - Static method in class org.biolag.particleprocessor.ParticlesAnalyizer
:   calc the center of mass of the particle

calcPrimeter(ArrayList<Particle>) - Static method in class org.biolag.particleprocessor.ParticlesAnalyizer


calcPrimeter2(ArrayList<Particle>, AImage) - Static method in class org.biolag.particleprocessor.ParticlesAnalyizer


calcProp(ArrayList<Particle>, double, double, double) - Static method in class org.biolag.particleprocessor.ParticlesAnalyizer
:   Proportionality of the contour area to a given typical particle's area

calcProp(ArrayList<Particle>) - Static method in class org.biolag.particleprocessor.ParticlesAnalyizer


centeralMoment(int, int, ArrayList<Point>) - Static method in class org.biolag.particleprocessor.Moments
:   Calculate the central moments

compactness(ArrayList<Point>) - Static method in class org.biolag.particleprocessor.Moments
:   Calculate the compactness of set of of points.

ConcavePoint - Class in org.biolag.particleprocessor


ConcavePoint(double, int, int, int) - Constructor for class org.biolag.particleprocessor.ConcavePoint


ConcavePoint(double, int, Point) - Constructor for class org.biolag.particleprocessor.ConcavePoint


counter - Static variable in class org.biolag.particleprocessor.ParticleProcessor


createDummyParticle(int, Point) - Method in class org.biolag.particleprocessor.Particle
:   For additional editing a dummy particle is an empty particle that the
    user creates.


## E

ellipticity(ArrayList<Point>) - Static method in class org.biolag.particleprocessor.Moments
:   Calculate the ellipticity of a set of points.


## F

findParticles(AImage, int) - Method in class org.biolag.particleprocessor.ParticleFinder
:   Find all particles in the input images given a minimum threshold size.


## G

getAR() - Method in class org.biolag.particleprocessor.ParticlesModel


getArea() - Method in class org.biolag.particleprocessor.Particle


getarea() - Method in class org.biolag.particleprocessor.ParticlesModel


getbestkernelSize(AImage, int, int) - Static method in class org.biolag.particleprocessor.ParticlesAnalyizer


getBoundingbox() - Method in class org.biolag.particleprocessor.Particle


getCircularity() - Method in class org.biolag.particleprocessor.Particle


getcircularity() - Method in class org.biolag.particleprocessor.ParticlesModel


getCompactness() - Method in class org.biolag.particleprocessor.Particle


getcompactness() - Method in class org.biolag.particleprocessor.ParticlesModel


getConcavePoint(int) - Method in class org.biolag.particleprocessor.Particle


getConcavePoint(ArrayList<ConcavePoint>, Point, Particle.CONTOUR\_TYPE) - Static method in class org.biolag.particleprocessor.ParticlesAnalyizer


getConcaves() - Method in class org.biolag.particleprocessor.Particle


getConcavness() - Method in class org.biolag.particleprocessor.ConcavePoint


getConnectedCurve(ConcavePoint, ArrayList<ConcavePoint>, ArrayList<ConcavePoint>) - Static method in class org.biolag.particleprocessor.ParticlesAnalyizer


getContour() - Method in class org.biolag.particleprocessor.Particle


getContour\_type() - Method in class org.biolag.particleprocessor.Particle


getContourPoint(int) - Method in class org.biolag.particleprocessor.Particle


getCurves() - Method in class org.biolag.particleprocessor.Particle


getellipciticiy() - Method in class org.biolag.particleprocessor.ParticlesModel


getEllipticity() - Method in class org.biolag.particleprocessor.Particle


getHeight() - Method in class org.biolag.particleprocessor.Particle


getheightAverage() - Method in class org.biolag.particleprocessor.ParticlesModel


getIndex() - Method in class org.biolag.particleprocessor.ConcavePoint


getIntensityArea() - Method in class org.biolag.particleprocessor.Particle


getMajorAxisAnchorPoint1() - Method in class org.biolag.particleprocessor.Particle


getMajorAxisAnchorPoint2() - Method in class org.biolag.particleprocessor.Particle


getMaxarea(ArrayList<Particle>) - Static method in class org.biolag.particleprocessor.ParticlesAnalyizer


getMaxx() - Method in class org.biolag.particleprocessor.Particle


getMaxy() - Method in class org.biolag.particleprocessor.Particle


getMeanarea(ArrayList<Particle>) - Static method in class org.biolag.particleprocessor.ParticlesAnalyizer


getMedianarea(ArrayList<Particle>) - Static method in class org.biolag.particleprocessor.ParticlesAnalyizer


getMinarea(ArrayList<Particle>) - Static method in class org.biolag.particleprocessor.ParticlesAnalyizer


getMinorAxisAnchorPoint1() - Method in class org.biolag.particleprocessor.Particle


getMinorAxisAnchorPoint2() - Method in class org.biolag.particleprocessor.Particle


getMinx() - Method in class org.biolag.particleprocessor.Particle


getMiny() - Method in class org.biolag.particleprocessor.Particle


getModearea(ArrayList<Particle>, double) - Static method in class org.biolag.particleprocessor.ParticlesAnalyizer


getModels(File, int, int, int, boolean) - Static method in class org.biolag.particleprocessor.ParticleProcessor
:   Return a `ParticlesModel` of the detected particle in the input image.

getModelsColored(File, int, int, HSBPixel, HSBPixel) - Static method in class org.biolag.particleprocessor.ParticleProcessor
:   Grains finding in colored images using math morphology approach

getModelsMathMorphology(File, int, int, int, boolean) - Static method in class org.biolag.particleprocessor.ParticleProcessor
:   Grains finding using math morphology approach

getModelsRGBImage(File, HSBPixel, HSBPixel, int) - Static method in class org.biolag.particleprocessor.ParticleProcessor
:   Detect the particle by using the color thresholding approach.

getOrientation() - Method in class org.biolag.particleprocessor.Particle


getPartcle(AImage, Point, Point) - Static method in class org.biolag.particleprocessor.ParticleProcessor


getParticles() - Method in class org.biolag.particleprocessor.ParticleFinder


getparticles() - Method in class org.biolag.particleprocessor.ParticlesModel


getPoint(int) - Method in class org.biolag.particleprocessor.Particle


getPoints() - Method in class org.biolag.particleprocessor.Particle


getPosition() - Method in class org.biolag.particleprocessor.Particle


getPrimeter() - Method in class org.biolag.particleprocessor.Particle


getprimeter() - Method in class org.biolag.particleprocessor.ParticlesModel


getProportionality() - Method in class org.biolag.particleprocessor.Particle


getProportionalitySum() - Method in class org.biolag.particleprocessor.ParticlesModel
:   The number of particles.

getSTDarea(ArrayList<Particle>, double) - Static method in class org.biolag.particleprocessor.ParticlesAnalyizer


gettotalProp(ArrayList<Particle>) - Static method in class org.biolag.particleprocessor.ParticlesAnalyizer


getWidth() - Method in class org.biolag.particleprocessor.Particle


getwidthAverage() - Method in class org.biolag.particleprocessor.ParticlesModel


getXModels(File) - Static method in class org.biolag.particleprocessor.ParticleProcessor
:   This is a general particles finder function that only smooth and
    threshold locally the source image then detect the particles.


## I

isDummy() - Method in class org.biolag.particleprocessor.Particle


isIsfixed() - Method in class org.biolag.particleprocessor.Particle


ispariclesinArea(AImage, Point, Point, ArrayList<Particle>) - Static method in class org.biolag.particleprocessor.ParticleProcessor


## M

moment(int, int, ArrayList<Point>) - Static method in class org.biolag.particleprocessor.Moments


Moments - Class in org.biolag.particleprocessor
:   The moments manipulator class.

Moments() - Constructor for class org.biolag.particleprocessor.Moments


## N

nomalizedCenteralMoment(int, int, ArrayList<Point>) - Static method in class org.biolag.particleprocessor.Moments
:   A normalized version of `Moments.centeralMoment(int, int, java.util.ArrayList)` function


## O

org.biolag.particleprocessor - package org.biolag.particleprocessor


orientation(ArrayList<Point>) - Static method in class org.biolag.particleprocessor.Moments
:   Calculate the orientation angle of a set of points


## P

Particle - Class in org.biolag.particleprocessor
:   A general class for managing particles.

Particle() - Constructor for class org.biolag.particleprocessor.Particle


Particle(ArrayList<Point>) - Constructor for class org.biolag.particleprocessor.Particle


Particle(Element) - Constructor for class org.biolag.particleprocessor.Particle
:   Create a particle from an XML `Element`.

Particle(Particle) - Constructor for class org.biolag.particleprocessor.Particle
:   Copy constructor creates a clone particle of the input.

Particle.CONTOUR\_TYPE - Enum in org.biolag.particleprocessor


ParticleFinder - Class in org.biolag.particleprocessor
:   The detector of the particles in binary images.

ParticleFinder() - Constructor for class org.biolag.particleprocessor.ParticleFinder


ParticleProcessor - Class in org.biolag.particleprocessor
:   Is a set of algorithm for detecting particles on grayscale and colored images.

ParticleProcessor() - Constructor for class org.biolag.particleprocessor.ParticleProcessor


ParticlesAnalyizer - Class in org.biolag.particleprocessor
:   Given a set of particles detected on a binary image, this class is responsible for analyzing them.

ParticlesAnalyizer() - Constructor for class org.biolag.particleprocessor.ParticlesAnalyizer


ParticlesAnalyizer.CALC\_METHOD - Enum in org.biolag.particleprocessor


ParticlesModel - Class in org.biolag.particleprocessor
:   A model class for I/O manipulating of the particles.

ParticlesModel(ArrayList<Particle>) - Constructor for class org.biolag.particleprocessor.ParticlesModel


ParticlesModel() - Constructor for class org.biolag.particleprocessor.ParticlesModel


ParticlesModel(Element) - Constructor for class org.biolag.particleprocessor.ParticlesModel
:   Create a ParticlesModel from an XML file.

perimeter() - Method in class org.biolag.particleprocessor.Particle
:   An accurate estimation of the particle perimeter.


## R

removeDuplicates() - Method in class org.biolag.particleprocessor.Particle
:   Remove duplicates points if found.


## S

setArea(double) - Method in class org.biolag.particleprocessor.Particle


setBoundingbox(Rectangle) - Method in class org.biolag.particleprocessor.Particle


setCircularity(double) - Method in class org.biolag.particleprocessor.Particle


setCompactness(double) - Method in class org.biolag.particleprocessor.Particle


setConcaves(ArrayList<ConcavePoint>) - Method in class org.biolag.particleprocessor.Particle


setConcavness(double) - Method in class org.biolag.particleprocessor.ConcavePoint


setContour(ArrayList<Point>) - Method in class org.biolag.particleprocessor.Particle


setContour\_type(Particle.CONTOUR\_TYPE) - Method in class org.biolag.particleprocessor.Particle


setDummy(boolean) - Method in class org.biolag.particleprocessor.Particle


setEllipticity(double) - Method in class org.biolag.particleprocessor.Particle


setHeight(double) - Method in class org.biolag.particleprocessor.Particle


setIndex(int) - Method in class org.biolag.particleprocessor.ConcavePoint


setIntensityArea(double) - Method in class org.biolag.particleprocessor.Particle


setIsfixed(boolean) - Method in class org.biolag.particleprocessor.Particle


setMajorAxisAnchorPoint1(Point) - Method in class org.biolag.particleprocessor.Particle


setMajorAxisAnchorPoint2(Point) - Method in class org.biolag.particleprocessor.Particle


setMaxx(int) - Method in class org.biolag.particleprocessor.Particle


setMaxy(int) - Method in class org.biolag.particleprocessor.Particle


setMinorAxisAnchorPoint1(Point) - Method in class org.biolag.particleprocessor.Particle


setMinorAxisAnchorPoint2(Point) - Method in class org.biolag.particleprocessor.Particle


setMinx(int) - Method in class org.biolag.particleprocessor.Particle


setMiny(int) - Method in class org.biolag.particleprocessor.Particle


setOrientation(double) - Method in class org.biolag.particleprocessor.Particle


setParticles(ArrayList<Particle>) - Method in class org.biolag.particleprocessor.ParticleFinder


setPoints(ArrayList<Point>) - Method in class org.biolag.particleprocessor.Particle


setPosition(Point) - Method in class org.biolag.particleprocessor.Particle


setPrimeter(double) - Method in class org.biolag.particleprocessor.Particle


setProportionality(double) - Method in class org.biolag.particleprocessor.Particle


setWidth(double) - Method in class org.biolag.particleprocessor.Particle


size() - Method in class org.biolag.particleprocessor.ParticlesModel


## T

toParticlesModel() - Method in class org.biolag.particleprocessor.ParticleFinder
:   Convert the particles to a `ParticlesModel`

toPolygon() - Method in class org.biolag.particleprocessor.Particle
:   Convert the particle contour to a polygon.

toString() - Method in class org.biolag.particleprocessor.Particle
:   In general this functions produces an XML entry of the particle.

toString() - Method in class org.biolag.particleprocessor.ParticlesModel


## V

valueOf(String) - Static method in enum org.biolag.particleprocessor.Particle.CONTOUR\_TYPE
:   Returns the enum constant of this type with the specified name.

valueOf(String) - Static method in enum org.biolag.particleprocessor.ParticlesAnalyizer.CALC\_METHOD
:   Returns the enum constant of this type with the specified name.

values() - Static method in enum org.biolag.particleprocessor.Particle.CONTOUR\_TYPE
:   Returns an array containing the constants of this enum type, in
    the order they are declared.

values() - Static method in enum org.biolag.particleprocessor.ParticlesAnalyizer.CALC\_METHOD
:   Returns an array containing the constants of this enum type, in
    the order they are declared.


## W

widthANDheight(ArrayList<Point>, ArrayList<Point>, ArrayList<Point>) - Static method in class org.biolag.particleprocessor.Moments
:   Calculate the width and height of a set of points

widthANDheight\_bruteforce(ArrayList<Point>, ArrayList<Point>, ArrayList<Point>) - Static method in class org.biolag.particleprocessor.Moments
:   Calculate the width and height of the particles by examining all points
    and finding the major and minor axes.

A C E F G I M N O P R S T V W

- Package
- Class
- Tree
- Deprecated
- Index
- Help

*org.biolag.particleprocessor 1.0*

- Prev
- Next

- Frames
- No Frames

- All Classes
